# Literature on Applied Machine Learning in Metagenomic Classification: A Scoping Review

**DOI:** 10.3390/biology9120453

**Published:** 2020-12-09

**Authors:** Petar Tonkovic, Slobodan Kalajdziski, Eftim Zdravevski, Petre Lameski, Roberto Corizzo, Ivan Miguel Pires, Nuno M. Garcia, Tatjana Loncar-Turukalo, Vladimir Trajkovik

**Affiliations:** 1 Faculty of Computer Science and Engineering, Saints Cyril and Methodius University, 1000 Skopje, Macedonia; slobodan.kalajdziski@finki.ukim.mk (S.K.); eftim.zdravevski@finki.ukim.mk (E.Z.); petre.lameski@finki.ukim.mk (P.L.); vladimir.trajkovik@finki.ukim.mk (V.T.); 2 Department of Computer Science, American University, Washington, DC 20016, USA; rcorizzo@american.edu; 3 Instituto de Telecomunicações, Universidade da Beira Interior, 6200-001 Covilhã, Portugal; impires@it.ubi.pt (I.M.P.); ngarcia@di.ubi.pt (N.M.G.); 4 Computer Science Department, Polytechnic Institute of Viseu, 3504-510 Viseu, Portugal; 5 Health Sciences Research Unit: Nursing, School of Health, Polytechnic Institute of Viseu, 3504-510 Viseu, Portugal; 6 Faculty of Technical Sciences, University of Novi Sad, 21102 Novi Sad, Serbia; turukalo@uns.ac.rs

**Keywords:** metagenomics, scoping review, classification, data preprocessing

## Abstract

**Simple Summary:**

Technological advancements have led to modern DNA sequencing methods, capable of generating large amounts of data describing the microorganisms that live in samples taken from the environment. Metagenomics, the field that studies the different genomes within these samples, is becoming increasingly popular, as it has many real-world applications, such as the discovery of new antibiotics, personalized medicine, forensics, and many more. From a computer science point of view, it is interesting to see how these large volumes of data can be processed efficiently to accurately identify (classify) the microorganisms from the input DNA data. This scoping review aims to give an insight into the existing state of the art computational methods for processing metagenomic data through the prism of machine learning, data science, and big data. We provide an overview of the state of the art metagenomic classification methods, as well as the challenges researchers face when tackling this complex problem. The end goal of this review is to help researchers be up to date with current trends, as well as identify opportunities for further research and improvements.

**Abstract:**

Applied machine learning in bioinformatics is growing as computer science slowly invades all research spheres. With the arrival of modern next-generation DNA sequencing algorithms, metagenomics is becoming an increasingly interesting research field as it finds countless practical applications exploiting the vast amounts of generated data. This study aims to scope the scientific literature in the field of metagenomic classification in the time interval 2008–2019 and provide an evolutionary timeline of data processing and machine learning in this field. This study follows the scoping review methodology and PRISMA guidelines to identify and process the available literature. Natural Language Processing (NLP) is deployed to ensure efficient and exhaustive search of the literary corpus of three large digital libraries: IEEE, PubMed, and Springer. The search is based on keywords and properties looked up using the digital libraries’ search engines. The scoping review results reveal an increasing number of research papers related to metagenomic classification over the past decade. The research is mainly focused on metagenomic classifiers, identifying scope specific metrics for model evaluation, data set sanitization, and dimensionality reduction. Out of all of these subproblems, data preprocessing is the least researched with considerable potential for improvement.

## 1. Introduction

Metagenomics is becoming an increasingly popular field in bioinformatics since with the evolution of technology and machine learning models, we are able to create increasingly more competent models to tackle the problems of DNA sequencing and genome classification. The genome is defined as the full genetic information of an organism, and genomics deals with obtaining the genome from a cultivated sample of a given organism. In contrast, metagenomics deals with samples from the environment that likely contain many organisms. The goal in this case is to analyze the different genomes within this environmental sample. Over the years, many Bacterial Artificial Chromosome (BAC) libraries have been gathered, even ones that can be used to sequence the entire human genome [1]; however, we were only able to tackle one genome at a time. With technology becoming more sophisticated, new more precise DNA sequencing techniques have been developed, and the computation power of modern computers has greatly increased. As a result of the latter, we can now process much larger quantities of data and train more complex machine learning models that were previously not feasible due to hardware limitations. This opens the gates for metagenomics to be one of the most trending topics in Big Data, as it can be used extensively in medicine. Such exemplary applications are in the identification of novel biocatalysts and the discovery of new antibiotics [2], as well as personalized medicine [3,4,5], bioremediation of industrial, agricultural, and domestic wastes [6,7], resulting in a reduction of environmental pollution, as well as forensics [8].

One example of a recent initiative in the field of forensics is the Critical Assessment of Massive Data Analysis (CAMDA) [9] Metagenomic Forensics Challenge, which encourages researchers to try and construct multi-source microbiome fingerprints and predict the geographical origin of mystery samples, given a large data set of city microbiome profiles in a global context (for example, the MetaSUBdata set [10]). An efficient and reliable model that can accurately determine the source of a microbiome fingerprint can be a valuable tool in forensics as it can allow us to answer many questions, for example: Where has a given individual been in the past six months given the microbiological sample obtained from the skin? However, to do this, we would have to deal with challenges, each of which offers much room for research on different parts of the problem:

1. Data science: How do we preprocess the data and deal with large quantities of unknown samples in the training set (erroneous data, unclassified DNA sequences, etc.)?
2. Big Data: How do we design a data pipeline to efficiently process exorbitant amounts of data samples, while not suffering extreme performance losses (model complexity vs. performance tradeoff)?
3. Machine learning: Which are the state-of-the-art models that can be used for metagenomic classification, and how can they be specialized towards the metagenomics domain?

These are all trending topics in the computer science research world, which is why it is to be expected that there is much related work out there. The goal of this study is to conduct a literature scoping review and discover work that can give potential answers to the questions presented above in terms of the CAMDA challenges. Ideally, we would like to analyze the trends from the past five years in data processing and metagenomic classification models to identify the current state-of-the-art approaches and discover their drawbacks. The ultimate goal is to narrow down the interesting research topics in this field for the foreseeable future. We would like to answer the following questions:

- What are the research trends about metagenomics in general, and what is being researched in terms of the CAMDA MetaSUB challenges?
- How do we efficiently deal with a large number of unknown samples within the data set from a data processing viewpoint?
- What are the current machine learning models that are suitable to be applied for the annual CAMDA MetaSUB challenges?

Based on these questions, we manually analyzed 11,764 articles, where Europe is the region with the most studies in the field of metagenomic sequencing with major collaborations with the United States and China. According to the different years of publication, “genome sequencing evaluation” is the most relevant search keyword.

The remainder of the review is organized as follows: Section 2 presents the methodology implemented. The results of the findings are presented in Section 3, and they are discussed in Section 4. This study is finalized in Section 5 with the main conclusions of the study.

## 2. Methods

This study adopts a scoping review method to identify and process the available literature. To this end, a Natural Language Processing (NLP) toolkit [11] is used to search the literature corpus. The toolkit follows the “Preferred Reporting Items for Systematic reviews and Meta-Analyses” (PRISMA) methodological framework [12]. The goal of this methodology is to identify and gather relevant articles based on certain criteria using some search keywords, sanitize the results by removing duplicates and other irrelevant or incomplete articles from the result set, and pick the articles that should undergo thorough screening after performing a qualitative analysis on the sanitized result set. The NLP toolkit automates this process and additionally provides a visual summary to report the results. This allows us to follow a methodological framework for conducting a scoping study [13] consisting of five stages: identification of the research question, identification of relevant studies, study selection, charting the data, and collating, summarizing, and reporting the results (Supplementary Materials Data S1).

### 2.1. Identification of Relevant Studies

In this stage, we specify the search parameters for the scoping study: Which digital libraries does our literature corpus consist of; what are the keywords we are looking for in an article; what is the publication time interval we are interested in, as well as other parameters of the NLP toolkit [11,14]? Currently, the toolkit indexes the following digital libraries: IEEE Xplore, Springer, and PubMed. All PubMed articles that match the given search criteria (i.e., a keyword) are analyzed. IEEE Xplore results include the top 2000 articles that match the given criteria, sorted by relevance determined by IEEE Xplore. For the Springer digital library, the search for each keyword separately is limited to 1000 articles or 50 pages with results (whichever comes first) sorted by relevance determined by Springer. The parameters that the toolkit requests as the input are the following:

- Keywords are search terms or phrases that are used to query a digital library. Duplicates that might occur in the results are removed in a later phase.
- Roperties are words or phrases that are searched in the title, abstract, or keywords section of the articles identified with the keywords.
- Property groups are thematically, semantically, or otherwise grouped properties for a more comprehensive presentation of the results.
- Start year indicates the starting year of publishing (inclusive) for the papers to be included in the study.
- End year is the last year of publishing (inclusive) to be considered in the study.
- Minimum relevant properties is a number denoting the minimum number of properties that an article has to contain to be considered as relevant.

For this scoping review, we would like to give a summary of the research work done in the field of metagenomic classification and data processing in the past ten years. The input parameters provided to the toolkit in our scoping review are shown in Table 1.

### 2.2. Study Selection and Eligibility Criteria

**Figure 1:**
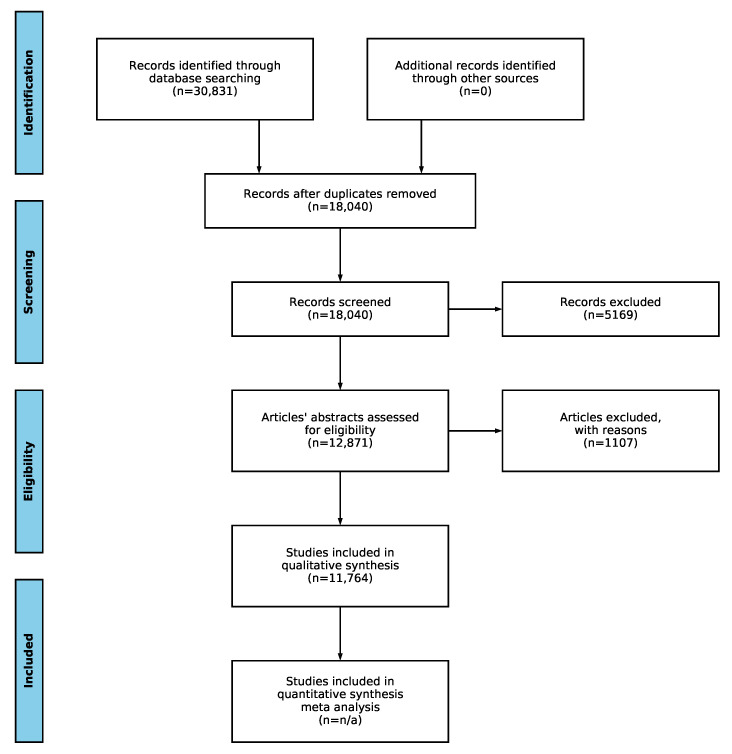
PRISMA statement workflow with total number of articles for the current survey.

After collecting the initial set of relevant studies, further filtering is done using the PRISMA methodology [12]. The workflow of the methodology is illustrated in Figure 1. The identification phase was already described above in Section 2.1. It is followed by the screening phase, which removes the duplicate articles, articles not published in the specified time period, and articles for which the title or abstract could not be analyzed due to parsing errors or unavailability. In the eligibility phase, the NLP toolkit applies natural language processing techniques to further filter the articles. In short, the toolkit takes the titles and abstracts of the articles and performs tokenization of sentences, English stop word removal, and stemming and lemmatization of the words [15]. Next, the stemmed and lemmatized words are matched against the set of properties that are given as input. If the article contains the required amount of relevant properties (in our case, 3, as shown in Table 1), the article is marked as relevant. The toolkit then automatically generates a BibTex file containing the citations to the relevant articles and an excel file containing the Digital Object Identifier (DOI), link, title, authors, publication date, publication year, number of citations, abstract, keyword, source, publication title, affiliations, number of different affiliations, countries, number of different countries, number of authors, BibTex cite key, number of found property groups, and number of found properties. This reduced set of relevant articles can then be analyzed further manually for potential inclusion in the qualitative and quantitative synthesis. While the manual part of the review cannot be avoided, the toolkit helps by reducing the domain of potentially interesting articles, making it easier for the researcher to find interesting articles relevant for the research topic, while reducing the number of irrelevant articles the researcher has to read through in the process.

### 2.3. Charting the Data

To visualize the results, the relevant articles are aggregated according to several criteria:

- source (digital library) and relevance selection criteria
- publication year
- digital library and publication year
- search keyword and digital library
- search keyword and year
- property group and year
- property and year, generating separate charts for each property group
- number of countries, distinct affiliations, and authors, aiming to simplify the identification of collaboration patterns (e.g., written by multiple authors with different affiliations).

These aggregated metrics are available in the form of CSV files and charts. The plotting of the aggregate results was integrated and streamlined using the Matplotlib library [16] and NetworkX [17]. The NLP toolkit enables graph visualization of the results, where the nodes are the properties, and the edges have weights determined by the number of articles that contain the two properties the edge connects. Articles that do not contain at least two properties and properties that were not present in at least two articles were excluded.

A similar graph for the countries of affiliations was generated. The top 50 countries by the number of collaborations were considered for this graph. Countries and an edge between them were shown if the number of bilateral or multilateral collaborations was in the top 10% (above 90th percentile) within those 50 countries.

## 3. Results

For this scoping review, it can be seen in Figure 1 that initially, 30,831 articles were identified in the database search phase. After the removal of duplicates, this number dropped to 18,040, which is almost half. Further screening reduced this number to 12,871. Applying the eligibility criteria described above left us with 11,764 articles to manually analyze, which was slightly over one-third of the initial articles discovered in the identification phase. We provide all the data generated by the NLP toolkit for reproduction purposes as supplementary material available at [18].

**Figure 2:**
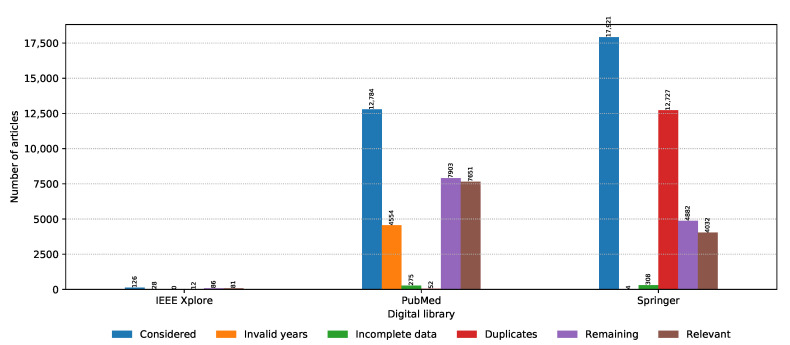
Number of articles collected and discarded in each PRISMA phase from each digital library.

**Figure 3:**
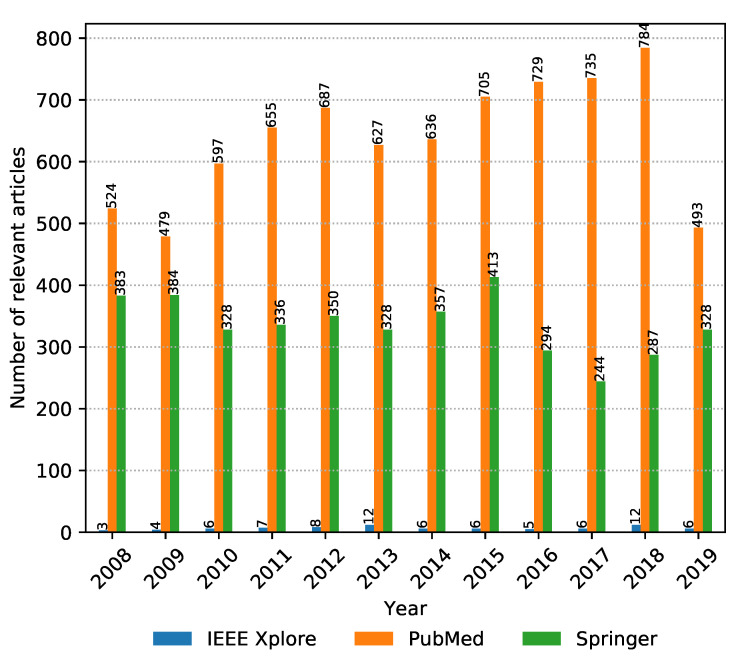
Number of relevant articles per year from 2008 to November 2019, aggregated by digital library source.

The numbers of collected articles, duplicates, articles with invalid time span or incomplete data, and relevant articles for each digital library are shown in Figure 2. The majority of articles considered are from PubMed and Springer with IEEE Xplore having negligible representation. Springer has the largest amount of initial articles; however, most of them are duplicates. In the end, the highest amount of relevant articles were drawn from PubMed. If we look at the number of relevant articles per year from each source presented in Figure 3, it can be seen that PubMed is consistently the prevalent digital library source when it comes to metagenomic sequencing; however, Springer was narrowing this gap in 2019. One additional thing to note in Figure 3 is that the data were collected on 8 November 2019, and as a result, the data for 2019 are incomplete. Even though only publications in the last two months of 2019 were not considered, due to the publication and indexing delays, this difference might be actually larger, and more articles published in 2019 would be excluded from the analysis. The same observation also applies to all other figures in the continuation of this section.

**Figure 4:**
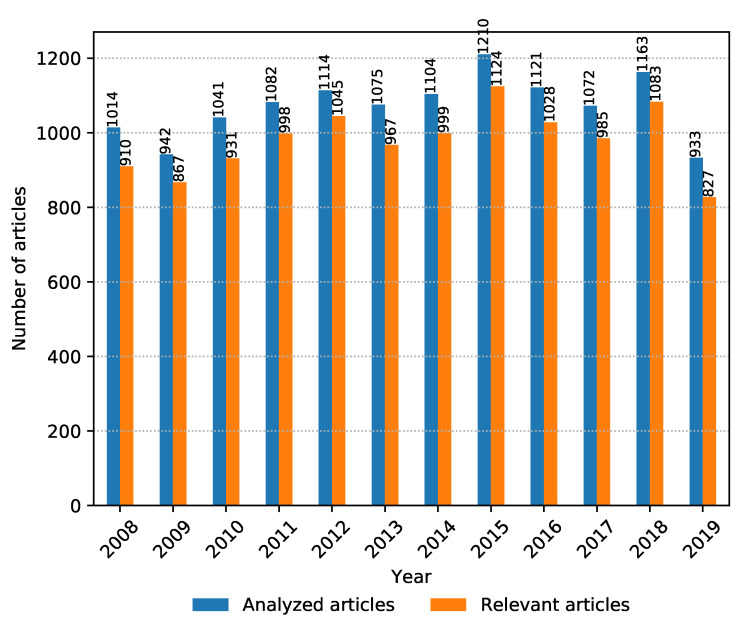
Number of collected and analyzed articles vs. number of relevant articles per year from 2008 to November 2019.

It is also interesting to see whether the total amount of relevant articles from all sources is increasing or decreasing over the years, which gives an idea of whether the research topic is becoming more or less trendy. The number of collected and relevant articles between 2008 and 2019 is shown in Figure 4. It can be seen that metagenomic sequencing has been a popular research topic in the past decade and continues to stay relevant going forward.

### 3.1. Geographical Distribution and Collaboration Evidence

**Figure 5:**
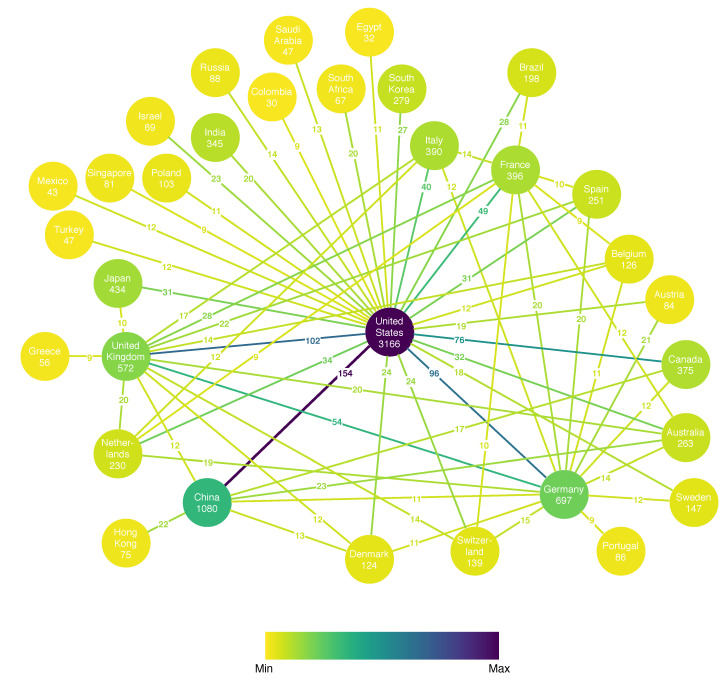
Number of research papers per country and collaboration links with the annotated number of joint articles.

Another point of interest is which countries are producing the most relevant papers in the field of metagenomic sequencing and whether collaboration between countries exists. The toolkit presents this information in a weighted property graph where the node properties include the country name and number of published articles discovered, while the edge attribute (weight) is the number of joint articles with authors from the two countries. For clarity, only the pairs with a number of collaborations greater than the 90th percentile are illustrated in Figure 5. The graph covers 32 countries (nodes) and 65 collaborations (edges). The stronger collaborations are color-coded with stronger ones in violet color, whereas weaker collaborations are pale. The same holds for the nodes and the number of articles. We can easily distinguish several hubs: the United States, China, Germany, the United Kingdom, Japan, France, and Italy with a large number of articles, as well as many collaborations with the rest of the hubs. An interesting observation is that while the United States is the biggest hub, if the European Union countries are aggregated together, they amount to a total number of 3262 articles, which outnumbers the U.S., making Europe the leader in research in the field of metagenomic sequencing. It is also interesting to notice that the U.S. is collaborating with China the most, while the EU countries collaborate more with the U.S. and China than among themselves.

### 3.2. Keyword Statistics

**Figure 6:**
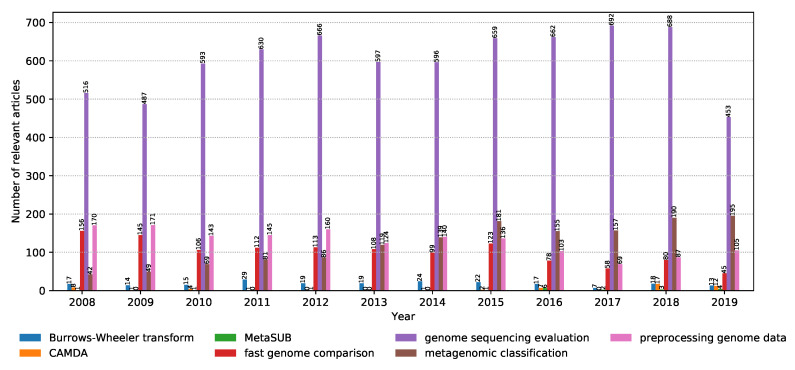
Distribution of the number of relevant articles for each keyword with respect to the publication year from 2008 to November 2019.

As already mentioned in Section 2.1, the articles were discovered using keywords (properties) to query the digital libraries. Metagenomic sequencing is a very broad field, and as a result, it is important to be able to identify the most trendy topics within the field. The distribution of relevant articles with respect to the publication year is shown in Figure 6. Note that the results for 2019 are truncated due to the analysis being done until November. In addition, the internals of their search engines are not known, meaning that the libraries might differ in the way they look for the keywords: only in the title, keywords section, abstract, or the whole article. It can be seen that the interest in genome sequencing is slightly increasing over the years, with a shift in interest from fast genome comparison to data preprocessing and model evaluation. This is in line with the rise of the popularity of the CAMDA challenges in the past two years, as well as the MetaSUB data set used in the CAMDA forensics challenge, where dealing with a large volume of data and adequate preprocessing is crucial to obtain good results.

**Figure 7:**
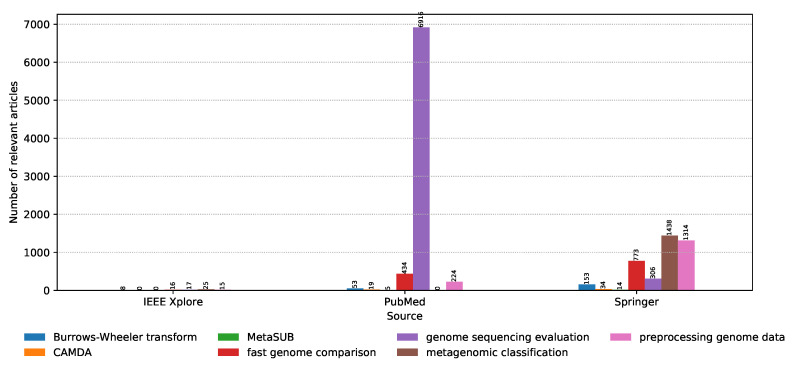
Distribution of the number of relevant articles for each keyword with respect to the digital library.

On the other hand, the distribution of relevant articles with respect to the digital library of publishing is shown in Figure 7. We have already seen that IEEE had an insignificant amount of relevant articles; however, it is interesting that the relevant articles from PubMed focus almost exclusively on model evaluation for genome sequencing, while Springer is more focused on the anatomy of the models, the classification process, as well as the data preprocessing techniques. In short, PubMed is more focused on the state-of-the-art approaches for constructing the data sets, while Springer is more focused on processing these data sets and constructing classifiers that work with them.

### 3.3. Property Statistics

**Figure 8:**
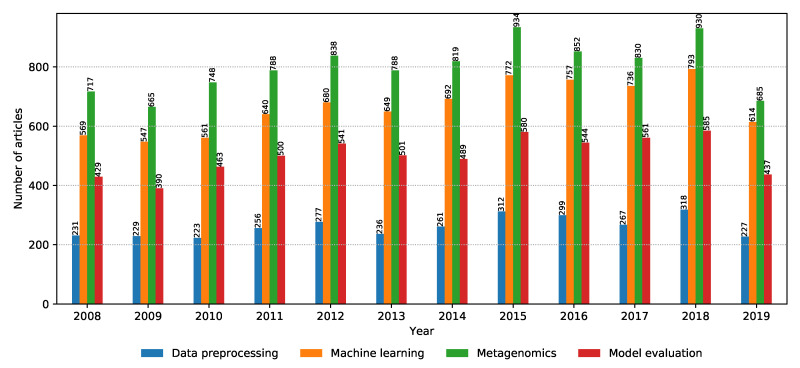
Annual distribution of the number of relevant articles for each property group.

Before going into deeper discussions for each property group, it is worth observing the annual distributions of relevant articles across property groups, shown in Figure 8. A slight increase in relevant articles can be noticed over the years; however, the relative difference between the number of relevant articles for the different property groups remains the same. With machine learning lately being at the center of attention in the computer science research world, it is a positive surprise that the metagenomics property group takes the lead with the most relevant articles consistently every year, indicating that bioinformatics is a greatly trending topic in computer science even outside of the machine learning domain. It is then followed by the property groups: machine learning, model evaluation, and data preprocessing, respectively. The low amount of relevant articles related to data preprocessing may indicate that there is much opportunity for novel ideas, explaining the motivation behind the CAMDA challenge where one of the biggest goals is to efficiently deal with large amounts of uncategorized data.

**Figure 9:**
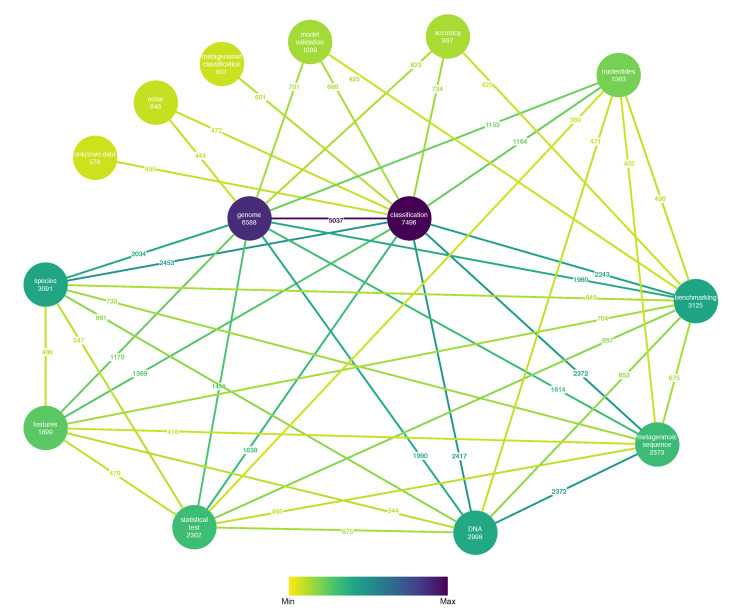
Number of relevant articles per property and number of co-occurrences of properties in relevant articles.

Furthermore, Figure 9 illustrates a weighted property graph with properties as nodes along with the number of relevant articles discovered containing each property and the number of co-occurrences in relevant articles between properties as edge weights. For clarity reasons, only the pairs with a number of co-occurrences greater than the 75th percentile are illustrated. Genome classification is clearly the most popular combination. It is worth noting that model benchmarking also appears to be a popular topic and is strongly linked with statistical tests.

## 4. Discussion

The following sections provide an in-depth analysis of the most recent research related to the property groups (metagenomics, machine learning, model evaluation, and data preprocessing). Each of the property groups has a dedicated section for discussing the latest trends, tools, and inventions that relate to our primary research topic: metagenomic classification.

### 4.1. Metagenomics

**Figure 10:**
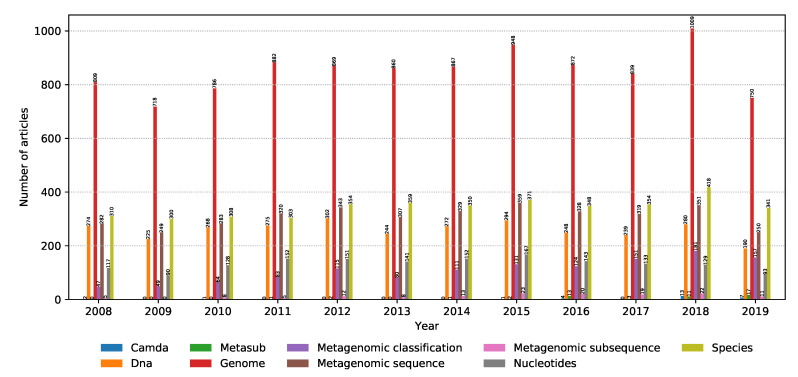
Number of relevant articles containing each property grouped into the metagenomics domain.

Evidence in the literature, as shown in Figure 10, shows that metagenomics as a field of study is becoming an increasingly popular research topic. The amount of relevant articles in the field is steadily increasing over the years as metagenomic analysis finds applications in many fields, including medicine [3], waste management [6], and forensics [8]. Computer science is not an exception, indicated by the high amount of relevant articles on metagenomic classification and DNA sequencing.

#### 4.1.1. Metagenomics in the Real World

The real-world applications of metagenomics are abundant. According to the work done in [19], research trends between 1996 and 2016 show that metagenomics is applied in the following fields (sorted in increasing order according to the number of publications per field): neuroscience, pharmacology, toxicology, chemistry and chemical engineering, mathematics, computer science, environmental science, agricultural and biological sciences, immunology and microbiology, medicine, biochemistry, genetics, and molecular biology. Most of the documented work (69.95%) is published as articles, followed by reviews and conference papers.

It is no surprise that medicine and biochemistry are the top fields, given that the roots of metagenomics hail from biology. There are applications of metagenomics in the human gut microbiome [20], where next-generation sequencing technology is used to study intestinal microbiome diversity and dysbiosis, leading to the identification of new functional genes, microbial pathways, and antibiotic resistance genes. However, the work also mentions that there are still some limitations including difficulties identifying microbial expression and the need for higher sequence coverage than the one provided by the 16S rDNA sequence analysis [21]. International projects studying the diversity of the human gut microbiome include the European project MetaHIT [22], and the American Human Microbiome Project [23].

Next-generation sequencing technology is also used for pathogen detection [24], as nearly all infectious agents contain DNA or RNA genomes. This raises the need for an optimized sequencing methodology that will allow the simultaneous and independent sequencing of billions of DNA fragments. Metagenomic Next-Generation Sequencing (mNGS) can be targeted towards microbial culture samples or untargeted. The untargeted approaches use shotgun sequencing [25] of clinical samples, whereas targeted approaches are based on singleplex or multiplex Polymerase Chain Reaction (PCR) [26], primer extension [27], or the more modern bait probe enrichment methods [28] to restrict detection to a list of targets. In summary, the study claims that mNGS has reduced the cost of high-throughput sequencing by several orders of magnitude since 2004. In addition, the work in [29] shows that mNGS approaches can be effectively used to assist the diagnosis of bloodstream infections using pathogen detection, while [30] shows that mNGS can be used to predict antibiotic and antiviral resistance.

On top of antibiotic resistance, metagenomics is also applied in field of pharmacy [31] where different techniques are used to understand the effect of antibiotics on microbial communities in order to synthesize new antibiotics that are highly effective against a target pathogen. In the absence of cultured microorganisms, metagenomics provides a strong alternative to research microbes and potentially finds their weaknesses. Techniques applied include descriptive metagenomics, where the goal is to describe the structure of the microbial populations, and functional metagenomics, where new antimicrobials can be found by analyzing the absence of certain pathogens in different environments.

#### 4.1.2. Metagenomics in the Context of CAMDA and MetaSUB

In addition to the clinical applications of metagenomics, the field is extensively researched in the Critical Assessment of Massive Data Analysis (CAMDA) [9] conferences. These conferences provide annual challenges in the field of metagenomics based on the MetaSUB data set of microorganism samples from various subways from all over the world. Works have been done to unravel bacterial fingerprints of city subways from microbiome 16S gene profiles [32,33,34] and to show that bacterial composition across different cities is significantly different. This is of crucial importance as it potentially allows us to deduce the location of a given sample, which can have many applications. For instance, we can train a model to identify where a person has been based on bacterial samples from his/her skin left over from the subways he/she was using.

One of the biggest challenges of the CAMDA conferences is to deal with extremely large quantities of data. Abundance-based machine learning techniques [35] have proven effective on the MetaSUB data set in the attempt to classify unknown samples. In this work, two approaches are proposed: the first one is a read-based taxonomy profiling of each sample, and the second one is a reduced representation assembly-based method. What is interesting to note is the fact that out of various machine learning techniques tested, random forests, in particular, have shown very good results with an accuracy of 91% with a 95% confidence interval between 80% and 93% on the read-based taxonomic profiling and 90% accuracy on the assembly-based model.

Another challenge is having a large amount of unlabeled data in the data set. One solution is the MetaBinG2model [36]. This model creates a reference database for caching and populates it with mappings between the unknown samples from the data set and their estimated “real” labels. The label estimation for a sequence of length k is obtained using a $k^{th}$-order Markov model with transition probabilities to all other possible sequences ($4^{k+1}$ in total given there are four nucleotides) based on sequence (k-mer substring) overlap. This model has been tested on GPUs for performance gains, and results show that a million 100 bp Illumina sequences can be classified in about 1 min on a computer with one GPU card.

While these are all significant findings, it is interesting to explore what else can be done with modern machine learning approaches in the scope of the CAMDA MetaSUB challenges. In the following sections, we investigate more deeply what can be done with various models in metagenomics, how to properly evaluate the models, and what can be done to clean the data before model training.

### 4.2. Machine Learning Techniques and Metagenomics

**Figure 11:**
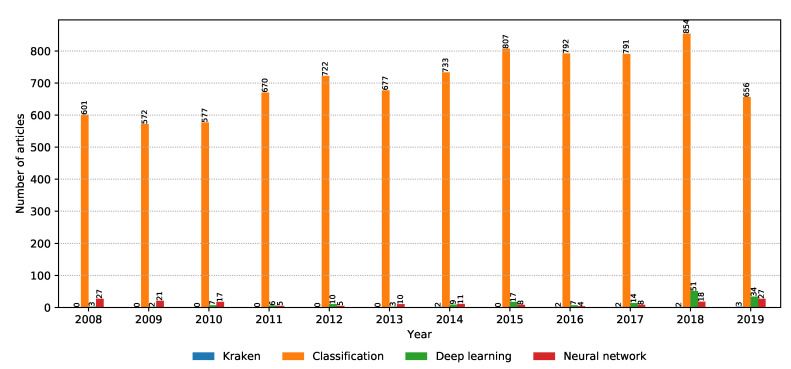
Number of relevant articles containing each property grouped into the machine learning domain.

Machine learning is the current buzz word in computer science, especially after the boom of deep learning and convolutional neural networks. The number of relevant articles for each property within the machine learning property group is shown in Figure 11. Classification is the main task in supervised machine learning, and it continues to be an interesting field of research going into the future. What is interesting is the increase in the popularity of deep learning and neural networks over the past two years, with a strong possibility of this trend to continue in the future. Next, we discuss the current machine learning trends in metagenomic classification that are picked up by this scoping review.

#### 4.2.1. K-mer-Based Approaches

The simplest approach to classify metagenomic sequences is the Kraken classifier [37]. The idea is simple and revolves around building a database to store the taxonomy tree of genomes. The database has records that map every k-mer (a subsequence of length k) to the Lowest Common Ancestor (LCA) in the taxonomy tree. To classify a sequence, a result set of LCAs is constructed for each k-mer in the sequence, after which a label is determined according to the number of hits. If no k-mers have a mapping in the database, the sequence is not assigned a class. This is a very basic lazy learning approach relying on a pre-built database and is used in many of the other works as a baseline for performance evaluation. It is worth noting that there have been several attempts to optimize the basic Kraken classifier, including KrakenUniq [38] (an adaptation of Kraken that contains an algorithm for assessing the coverage of unique k-mers found in each species in a data set) and LiveKraken [39] (real-time classifier based on Kraken).

In the previous section, it was mentioned that ensemble models, in particular random forests, have proven to be quite effective in the CAMDA MetaSUB challenges. This is not a novel idea as random forest-based classifiers for metagenomic sequences [40,41] date back to as early as 2013. These approaches apply binning techniques to fragments obtained through the Whole Genome Shotgun (WGS) sequencing. However, these methods are now outdated and outperformed even by the simple Kraken classifier. A more modern solution uses deep forests based on the phylogenetic tree [42]. Namely, this approach extends a standard deep forest model by embedding phylogenetic tree information to obtain the so-called cascade deep forest. Each cascade consists of two parts: random forests and completely random forests. Completely random forests are defined such that the features chosen for each split are taken randomly until all features are used, as opposed to random forests where splits are chosen to maximize information gain using some metric (entropy, Gini index, etc.). Each of the forests produces a class vector, which together with the original feature vector is forwarded as input to the next cascade. By connecting multiple cascades, we obtain the deep random forest. We can add as many cascades as we see fit considering the complexity of the task at hand, and this way, we define a structure similar in concept to Deep Neural Networks (DNNs). Furthermore, the cascades can be flattened out to reduce the number of decision trees per cascade and increase the depth. The authors claimed that the performance of this model is competitive with DNNs with the following advantages: (1) it can adaptively determine the depth of the model; (2) there are fewer parameters to tune.

Another approach is the MetaNN model [43] based on deep neural networks. This model is implemented in the following pipeline:

1. Extract the raw data set;
2. Filter out microbes that appear in less than 10% of the total samples for each data set (preprocessing);
3. Create an augmented data set using a Negative Binomial (NB) distribution to fit the training data, and then, sample the fitted distribution;
4. Train a DNN on the augmented training set (multilayer perceptron or convolutional neural network).

When using a Multilayer Perceptron (MLP), the authors used two to three hidden layers to avoid over-fitting of the microbial data. When using a Convolutional Neural Network (CNN), the authors arranged the bacterial species based on their taxonomic annotation, ordered them alphabetically, and concatenated their taxonomies (phylum, class, order, family, and genus). As a result, the CNN is able to extract the evolutionary relationships based on the phylogenetic sorting. The authors claimed that their model outperforms several other popular classification models including Support Vector Machines (SVM), random forest, gradient boosting, logistic regression, and multinomial naive Bayes. Alternative classification approaches adopt one-class classification methods such as isolation forest, Local Outlier Factor (LOF), and mixed ensembles [44,45]. These methods appear particularly useful in challenging domains that present extremely imbalanced data sets, such as those characterized by almost only negative data sequences.

#### 4.2.2. Non-K-mer-Based Approaches

One of the biggest weaknesses of k-mer-based classifiers is the weak scalability for large amounts of data [46], as the size of the data set influences both the training time and the accuracy of the models. To combat this, some approaches try to utilize feature extraction to simplify the data set and reduce dimensionality. One such approach is the use of Non-negative Matrix Factorization (NMF) [47]. The main idea is to represent the data points as a linear combination of non-negative features that can be computed from the data. Namely, a non-negative p×n matrix *X* can be approximated by TW, where *T* is a non-negative p×k matrix called the type (feature) matrix and *W* is a non-negative k×n weight matrix. If *k* is chosen such that (p+n)×k<<np, the dimensionality of the data is significantly reduced. In the case of metagenomics, the *X* matrix counts the occurrences of genes in microbes, i.e., $X_{ij}$ is the number of observations of gene *i* in sample *j*. This approach provides dimensionality reduction similar to Principal Component Analysis (PCA). The weight matrix *W* can then be calculated via non-negative Poisson regression of each sample in *X* on *T*. The weight matrix can then be used in a supervised classifier; however, it can also be applied in unsupervised learning approaches.

Another approach is a lightweight metagenomic classifier using an extension of the Burrows–Wheeler Transform (eBWT) [48]. Similar to the previous approach, this model takes advantage of the combinatorial properties of the eBWT to minimize internal memory usage required for the analysis of unknown sequences. The approach is alignment- and assembly-free (does not perform subsequence aligning) and compares each unknown sequence in the sample to all known genomes in a given collection. As a result, the approach is very lightweight compared to k-mer approaches while providing competitive classification results.

Other non-k-mer-based approaches [49,50,51] perform link prediction for GRN (Gene Regulatory Network) reconstruction on homogeneous networks, where the known existing gene interactions are considered as positive labeled links and all the possible remaining pairs of genes as unlabeled links. These methods exploit predictive models to classify unknown gene regulations. On the other hand, reference [52] performed link prediction on heterogeneous networks with the aim to detect associations between ncRNAs and diseases.

### 4.3. Model Evaluation

**Figure 12:**
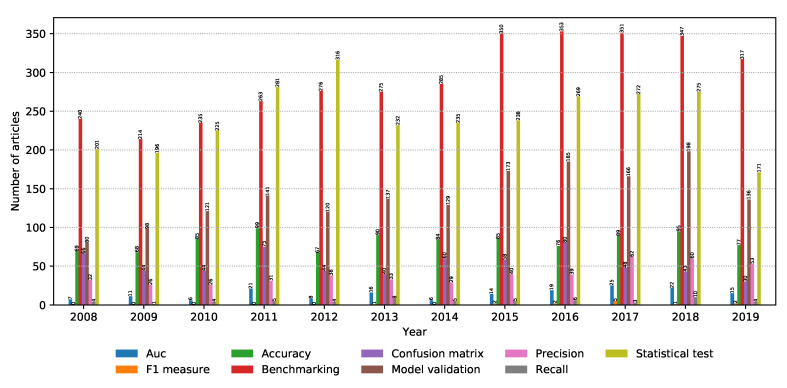
Number of relevant articles containing each property grouped into the model evaluation domain.

Finding the right classification model is just part of the job. The model needs to be evaluated in a realistic manner, given the scope of the data. This section focuses on modern model evaluation techniques in the metagenomics field. The number of relevant articles for each property within the model evaluation property group is shown in Figure 12. It can be seen that model evaluation and benchmarking are very hot topics, while statistical tests are a very popular tool to conduct model evaluation.

#### 4.3.1. Model Validation Metrics

To begin model evaluation, we first need a metric of quality of the model. Popular metrics include:

- Accuracy: the percentage of correct classifications on the testing set ($ACC=\frac{TP+TN}{P+N}$);
- Precision (positive predictive value): the percentage of true positives from all positively classified samples ($PPV=\frac{TP}{TP+FP}$);
- Recall (sensitivity or true positive rate): the percentage of true positives from all positive samples ($TPR=\frac{TP}{TP+FN}$);
- Specificity (selectivity or true negative rate): the percentage of true negatives from all negative samples ($TNR=\frac{TN}{TN+FP}$);
- F1 score: a metric taking both precision and recall into account ($F_{1}=2\frac{PPV*TPR}{PPV+TPR}$).

These metrics can be calculated multiple times with different training and test partitions of the data set. Usually, the best approach is to use stratified k-fold, where the data set is split into *k* partitions with equal class distributions, then the training is done *k* times with a different fold being used for testing each time. This way, we obtain *k* values for each performance metric and can calculate the mean values, after which we can use the *t*-test to see if the obtained mean is statistically significant or not.

While these metrics are very useful for model evaluation, they are very generic (they can be used to assess any machine learning model). The best scenario is if these metrics can be complemented by additional domain-specific metrics for the problem at hand. In the case of metagenomics where we are looking at DNA sequences, bit alignment scores are the most popular. In the case of the FunGAPgenome annotation pipeline [53], several bit alignment scores are aggregated and used for evaluating the model: Pfam [54], Benchmarking Universal Single-Copy Orthologs (BUSCO) [55], and BLAST [56]. These are combined to calculate the so-called evidence score using the following formula:

(1) Evidencescore=(BLASTscore∗coverage)+BUSCOscore+∑Pfamscores

BLAST is the oldest of the three and generates a score based on bit overlap between the query sequence and a database(s) of know sequences. It is used as a basis of other more sophisticated alignment tools such as MEGAN [57] and the already mentioned Kraken classifier. Pfam is more sophisticated and uses seed alignments to construct the database of protein domain families. It is designed with incremental updating in mind, meaning that when new sequences are released/discovered, it is very easy to add them to the database. BUSCO is an open-source tool that provides a quantitative assessment of genome assembly and annotation completeness based on evolutionarily informed expectations of gene content. It is capable of picking up complete or partial (fragmented) matches between input query sequences and the database of known sequences.

#### 4.3.2. Benchmarks

After defining the metrics that can be used for model performance measuring, we need something with which to compare the values. As already mentioned, Kraken is one simple model that is a good baseline for model evaluation. Alexa B. R. McIntyre, Rachid Ounit et al.provided us a benchmark for performance evaluation of 11 metagenomic classifiers in their work “Comprehensive benchmarking and ensemble approaches for metagenomic classifiers” [58]. The tests were done on a large data set containing the data of 846 species. The classifiers in question have different classification strategies:

1. K-mer-based (CLARK [59], CLARK-S [60], Kraken, Kraken_filtered, LMAT [61], naive Bayes classifier)
2. Alignment-based (BlastMegan_filtered, BlastMegan_filtered_liberal, DiamondMegan_filtered, MetaFlow [62])
3. Marker-based (GOTTCHA [63], METAPhlAn [64], PhyloSift [65], PhyloSift_filtered)

The models are evaluated on classifications on the genus, species, and subspecies level. The metrics used to evaluate the extent of the problems caused by false positives are the Area Under the Precision-Recall curve (AUPR) and the $F_{1}$ score. According to the mean AUPR (mAUPR), all tools perform best at the genus level (45.1%≤mAUPR≤86.6%), with small decreases in performance at the species level (40.1%≤mAUPR≤84.1%). Calls at the subspecies (strain) level show a more marked decrease in all measures for the subset of 12 data sets that include complete strain information (17.3%≤mAUPR≤62.5%). It is interesting to note that for k-mer-based tools, adding an abundance threshold increases the precision and F1 score, making them competitive with the marker-based tools.

The study also showed that the tools can be combined pairwise in an ensemble to further increase precision in taxonomic classification. In this manner, the pair between GOTTCHA and Diamond-MEGAN and BLAST-MEGAN paired with either Diamond-MEGAN, naive Bayes classifier, or GOTTCHA achieve precisions over 95%, while the other 24 pairs achieve precisions over 90%. In addition, memory consumption is also benchmarked by measuring maximum memory usage and time to load files into memory with respect to the file size. The results show that Clark, Clark-S, and Kraken seem to be the most memory-intensive, while PhyloSift and METAPhlAn seem to be the most memory efficient.

**Figure 13:**
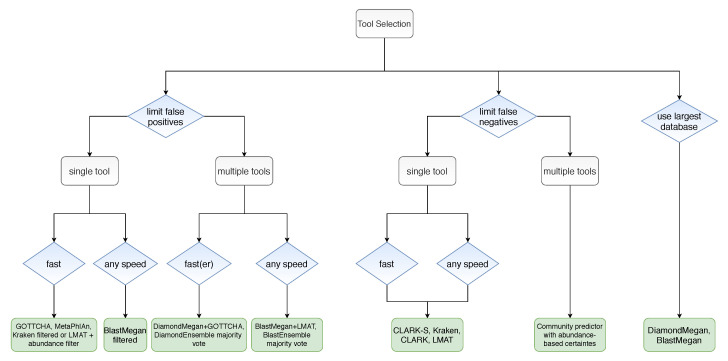
Algorithm for tool selection.

All things considered, the authors provided us with the decision tree shown in Figure 13, which can help us choose a suitable model, given the problem we want to solve and the constraints that we have. We can follow the tree by deciding whether our priority is to decrease the false-positive rate, decrease the false-negative rate, memory efficiency when using large databases, using a single model or an ensemble of pairs, faster or slower processing time, etc.

These benchmarks can be used for model valuation for any model, as they give us some performance metrics for comparison. While these benchmarks take various kinds of models based on different approaches into consideration and expose their strong and weak points, they do not consider one important thing: data quality. The next section explores ways to clean the input data.

### 4.4. Data Preprocessing

In practice, it has been shown many times that the difference between a clean data set and a dirty one can affect the final results more than a bad choice of a classifier. While the optimal situation is to have clean data with the minimal relevant features and the best model for the task at hand, many would argue that the former is even more important than the latter. Hence, fields like data mining and data science are very important in the machine learning world. These disciplines put much emphasis on the so-called data preprocessing step before we even start looking into choosing the right model for the job [66]. Data preprocessing steps include, but are not limited to:

- Dealing with missing data in the form of missing features or class labels (removing records with missing data or replacing missing data by best effort speculation about the missing value);
- Removing redundant features (features that contribute a little to the class variance and/or features that are strongly correlated or derived mathematically from other features) [67];
- Data discretization (converting continuous data into discrete values);
- Removing records with outlier values for certain features;
- Data sampling if the data set is too big to process [68].

**Figure 14:**
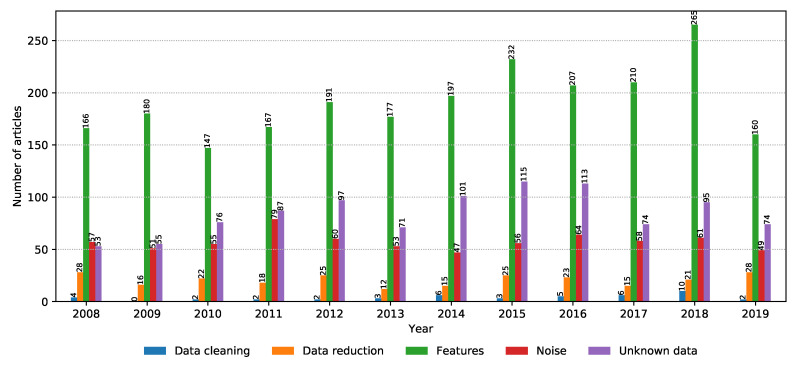
Number of relevant articles containing each property grouped into the data preprocessing domain.

Data curation is no simple task, and entire research projects are dedicated to cleaning data and extracting relevant data from the data set in order to reduce model training time and complexity [68]. The situation is no different in the field of metagenomics. The number of relevant articles for each property within the data preprocessing property group is shown in Figure 14. It can be seen that feature extraction, dealing with unknown data, and data reduction are all popular topics, with feature extraction becoming increasingly relevant since 2018.

#### 4.4.1. Dealing with Unknown (Unidentified) Sequences

One of the biggest issues with the large MetaSUB data set is the fact that there are many unknown (unidentified) sequences. These kinds of discrepancies happen for various reasons, the biggest one being imprecise measurements and faulty measuring equipment. In the case of metagenomics, this problem happens as a result of erroneous DNA sequencing when applying NGS techniques. An attempt to combat this problem is the Sequencing Error Correction in RNA-sequences (SEECER) [69] tool. It is based on Hidden Markov Models (HMMs). This method is capable of performing error correction of RNA-Seq data without the need for a reference genome. The method can cover non-uniform coverage and alternative splicing and has been shown to outperform other similar error correction methods on diverse human RNA-Seq data sets.

Another tool for dealing with unmapped sequences is DecontaMiner [70]. Compared with SEECER, this tool does not actually correct the errors, and it needs a reference genome to work; however, it can help us discover contaminating sequences that might be causing the sequencing errors leading to unknown sequences. Contaminating sequences can be ones from bacteria, fungi, and viruses. DecontaMiner runs on the data set and produces a visualization of the summary statistics and plots using D3 JavaScript libraries. This tool can be integrated easily in a data cleaning pipeline as a step to find potential contamination in the data, after which measures can be taken to reduce the sources of contamination. It may be interesting if any of these tools can be used on the MetaSUB data set to reduce the number of unknown sequences to improve classification performance.

#### 4.4.2. Feature Extraction and Data Reduction

In addition to cleaning the data set of erroneous data, it is also very useful to reduce the data set by removing data that are redundant, i.e., data that do not provide meaningful information to the classifier [66]. This can be a double-edged sword: if the data set is too big, it can increase model training time without providing better testing performance as a result of the larger data input; however, by removing too much data, we can end up with a data set that is too small and is not representative (bias has been introduced due to poor data reduction).

In metagenomics, the data set consists of the reads obtained from NGS. One way to reduce the data is to filter the data set to include only sequences from a given list of species. This can be done with the tool MetaObtainer [71]. The authors claimed that their tool works well on short reads, which is the biggest shortcoming of other similar tools. In addition, the list of sequences we want to filter does not necessarily have to contain known species; it can also find unknown species using reference genomes of species similar to the query sequence.

On top of reducing the data set, we can also reduce the dimensionality of the data, which can drastically speed up training for some models. This can safely be done for redundant features, i.e., features that do not give meaningful information about the class, are correlated, or are derived from other features in the data set. Amani Al-Ajlan and Achraf El Allali proposed a methodology [72] for feature selection using maximum Relevance Minimum Redundancy (mRMR) to find the most relevant features. The feature extraction algorithm has shown good results in improving classification results from Support Vector Machine (SVM)-based models. Another feature extraction algorithm [73] was proposed for efficient metagenomic fragment binning. Binning is the process of grouping random fragments obtained from WGS data into groups. The algorithm uses sub-sequence blocks extracted from organism protein domains as features. Binning predictions are then made using a classifier, such as a naive Bayes classifier or a random forest. Besides feature extraction, dimensionality can be reduced via other techniques such as the popular eigenvector-based Principal Component Analysis (PCA) and the above-mentioned NMF.

To conclude, although it will not improve model training time or reduce dimensionality, compressing the data set in some way may be useful to store large databases if we have limited disk memory. One tool that can do CRAM-based compression in parallel, while also providing the user with taxonomic and assembly information generated during compression, is MetaCRAM [74]. The tool provides reference-based, lossless compression of metagenomic data while boasting two to four-fold compression ratio improvements compared to gzip. The authors claim that the compressed file sizes are 2–13% of the original raw files.

## 5. Conclusions

This paper presents an overview of the state-of-the-art machine learning tools and techniques for metagenomic classification from the last decade. Topics covered include general novelties in the field of metagenomics, metagenomic sequencing, applied metagenomics in the CAMDA/MetaSUB challenges, efficient machine learning models, model evaluation, and data preprocessing techniques. In a way, this study provides an evolution timeline of metagenomic related computer science research.

Our methodology applies the NLP toolkit to search three digital libraries: PubMed, IEEE, and Springer for relevant papers in the domain of research. The toolkit takes keywords organized as properties and property groups as input and is configurable with respect to how many properties need to be present in the publication for it to be considered relevant. The tool later uses the PRISMA methodology to filter the results down to the core relevant research work. The property groups used for this scoping review were the following (sorted in descending order according to the number of relevant articles found): metagenomics, machine learning, model evaluation, and data preprocessing. After a manual examination of the relevant articles, it can safely be said that insightful information was obtained related to all the property groups.

This study confirms that metagenomics is a very hot topic in bioinformatics. More and more people are joining in on the CAMDA organized challenges for metagenomic data processing. The field is very broad, and all property groups represent valid subfields with a lot of potential for further research, whether it is trying out different new classifiers on the data to get better performance or solving the problems from a Big Data point of view by sanitizing the database, figuring out how to handle metagenomic unknown sequences in the data set, or reducing the dimensionality of the data. In all of these fields, we have baseline research that can be used as a starting point and then improved upon incrementally, and the community seems to be very active and involved in continuing the trend of organizing further machine learning challenges related to metagenomics.

One obvious shortcoming of this study is that the scoping is limited to three digital libraries. Even though these libraries are very popular and rich with content, potentially interesting research material may be located elsewhere. Furthermore, the technicalities of the search engines of the three libraries remain unknown (amount and format of search results returned may differ). As a result, the search queries used to obtain the results are the same for all platforms (no customization was done to try to optimize the search results). All in all, the scoping review yielded a large number of relevant articles, many of which provided us with truly insightful information, which is the main point of the review.

## Figures and Tables

**Table 1:** NLP toolkit input parameters for the metagenomic sequencing scoping review.

Input Parameter	Value
Keywords	CAMDA, MetaSUB, metagenomic classification, preprocessing genome data, deep learning, lightweight model, fast genome comparison, k-mers, Burrows–Wheeler transform, genome sequencing evaluation, model benchmarking
Property groups(properties)	metagenomics (metagenomic sequence/DNA sequence, species/organism/bacteria, metagenomic subsequence/k-mer, metagenomic classification/metagenomic sequence classification/metagenomic sequencing, genome/genetic material/DNA genome, nucleotides/monomer, DNA/deoxyribonucleic acid, CAMDA/CAMDA Pub, MetaSUB/forensics challenge)
	machine learning (classification/sequencing/categorization, deep learning/deep model/DL, neural network/deep neural network/DNN, deep forest, Kraken)
	data preprocessing (unknown data/unknown sequence/incomplete data, noise/white noise/error, data reduction, features/feature extraction, Big Data, data cleaning)
	model evaluation (performance, F1 measure, false positive/false-positive, accuracy, benchmarking, model validation, *T*-test/student test/statistic test)
Start year	2008
End year	2019
Minimum relevant properties	3

## Supplementary Materials

Data S1: We provide all data used for generating these figures online at https://zenodo.org/record/4289228#.X71rls1KguV, so that any interested researchers can use it to generate charts with a better quality.

## Abbreviations

The following abbreviations are used in this manuscript:

AUPR	Precision-Recall Curve
BAC	Bacterial Artificial Chromosome
BUSCO	Benchmarking Universal Single-Copy Orthologs
CAMDA	Critical Assessment of Massive Data Analysis
CNN	Convolutional Neural Network
DNN	Deep Neural Network
DOI	Digital Object Identifier
eBWT	extension of Burrows–Wheeler Transform
HMM	Hidden Markov Model
LCA	Lowest Common Ancestor
MLP	Multilayer Perceptron
mRMR	Maximum Relevance Minimum Redundancy
NB	Negative Binomial
NGS	Next-generation Sequencing
NLP	Natural Language Processing
NMF	Non-negative Matrix Factorization
PCA	Principal Component Analysis
PCR	Polymerase Chain Reaction
PRISMA	Preferred Reporting Items for Systematic Reviews and Meta-analyses
SEECER	Sequencing Error Correction in RNA-sequences
SVM	Support Vector Machine
WGS	Whole Genome Shotgun
